# Baby Sleep Project Protocol: a realist evaluation of an intervention to reduce preventable infant mortality

**DOI:** 10.1136/bmjopen-2024-091414

**Published:** 2025-02-13

**Authors:** Anna Pease, Becky Lambert, Jenny Ingram, N Bradley, Peter Fleming, Peter S Blair, Michelle Farr

**Affiliations:** 1 Population Health Sciences, University of Bristol Medical School, Bristol, UK; 2 Department of Marie Curie Palliative Care Research, University College London, London, UK; 3 NIHR Applied Research Collaboration West, Bristol, UK

**Keywords:** Health Education, Person-Centred Care, PUBLIC HEALTH, QUALITATIVE RESEARCH

## Abstract

**Abstract:**

**Introduction:**

In the UK, approximately 300 infants each year die suddenly and unexpectedly, with most deaths remaining unexplained. Population-wide ‘Safer Sleep’ messages have brought rates down but remaining deaths now predominantly occur within families experiencing poverty. Many of these deaths may be preventable as the majority have known, avoidable risks present. New resources and tools for health professionals and families have been developed to address modifiable barriers to implementing safer sleep advice in this priority group of families. This study aims to understand how the Baby Sleep Project resources work to improve the uptake of safer sleep advice, including for whom, and in what contexts they work best.

**Methods and analysis:**

Realist evaluation will be used, including both qualitative and quantitative methods. Data will be collected both pre- and post-health professional training in the new resources. We will invite neonatal staff, health visitors and family nurse partnerships nurses, and primary caregivers of infants to take part. We will carry out qualitative interviews with health professionals and caregivers. Quantitative surveys looking at implementation for health professionals, changes in infant care knowledge and practice, and parenting self-efficacy will be conducted with caregivers. Mechanisms of action, contexts and outcomes from the new resources will be tested against the initial programme theory. The findings from this research will inform evidence-based explanations of how to improve the uptake of health advice in priority populations.

**Ethics and dissemination:**

The study was given a favourable opinion by the South West—Frenchay Research Ethics Committee (ref: 23/SW/0119). We will publish our findings in academic journals and talk about them at conferences. We will make sure the people who took part in the study hear about them first. If the resources are shown to be useful, we will work with charities and the National Health Service to roll them out across the whole of the UK.

**Trial registration number:**

https://www.isrctn.com/ISRCTN73364337

**STRENGTHS AND LIMITATIONS OF THIS STUDY**

- A realist approach to the uptake of safer sleep messages across a priority population provides an opportunity for a deeper understanding of the mechanisms of advice giving for this group.
- Incorporating different sources of evidence using an iterative process will give insights beyond more traditional research (trial based) approaches.
- The study uses a pre- and post-training design to attempt to capture changes in infant care knowledge, practice and parental self-efficacy.

## Introduction

 Annually in the UK, 300 infants die suddenly and unexpectedly (sudden unexpected death in infancy (SUDI)).^1^ Of these deaths, most (approximately 70%) remain unexplained following investigation, labelled sudden infant death syndrome (SIDS) or unascertained. The risk factor profiles between SIDS and explained SUDI are similar, suggesting common pathways, and that interventions could potentially have an impact on both.^2 3^ Every death profoundly affects family members, health professionals and wider society.^4 5^ While public health messages for safer sleep have worked well in the general population, currently many deaths occur in families experiencing deprivation.^6 7^ Recent data from the National Child Mortality Database (NCMD) show a significantly larger proportion of unexplained deaths among families living in the most deprived neighbourhoods (42%), five times higher than those in the least deprived neighbourhoods (8%). Many deaths may be preventable as they involve modifiable risks within the infant’s sleeping environment.^8^ The population-level advice is failing to translate into impact for these families and there is a need for a better approach.^9^

Research into preventing unexpected and unexplained infant deaths reduced rates in the population of England & Wales to a low in 2021 of 0.27 per 1000 live births.^10^ Babies born to those in lower socio-economic groups remain at disproportionately higher risk, with rates for 2021 at 0.38 per 1000 live births for babies born into the lowest socio-economic classification.^10^ NCMD data from 2020 found that for 72% of the deaths at least one known sleep environment risk factor was present.^11^ In 2020, members of our team were commissioned by the National Safeguarding Panel Practice Review into SUDI to conduct a systematic review of the literature on interventions to prevent SUDI in families with children at increased risk of abuse or neglect.^12^ This work included a systematic review and thematic synthesis of qualitative research investigating decision-making for the infant sleep environment in families with children considered to be at risk of SUDI.^13^ This review highlighted the influence of ‘out of routine’ circumstances, and the role of peers and family in decision-making. Increased likelihood of risky infant sleep environments due to disruption to routine is also seen in epidemiological data. Unaccustomed sleeping environments are consistently seen to increase risk, where they are measured.^14^,^16^ While social *deprivation* is easily measured and correlates with increased risks for a range of diseases and outcomes, the mechanism by which social *disruption* mediates this relationship remains unclear. The development of effective new theory-based tools and resources is now essential to bring down death rates in those infants most at risk.

In the UK, health professionals, including health visitors, midwives, neonatal staff, support workers and specialist nurses, deliver safer sleep advice. They generally aim to increase parent knowledge of SUDI risk and protective factors. This model has worked well for some but can fail those starting from a disadvantage with multiple risk factors present at birth. Our previous work with health professionals suggests they would welcome a new approach for use with families with infants considered to be at increased risk.^17^,^19^ We have good data on which babies are more at risk,^20 21^ and growing evidence of several principles that can be actioned into a set of resources by families and health professionals to improve the credibility of advice, make safer sleep the priority during times of disruption and empower families with information and support to influence their decision-making and their own needs for sleep.^13 22 23^

Our aim in the Baby Sleep Project is to evaluate an intervention, comprising a suite of resources, to increase the uptake of safer sleep advice in families with infants at increased risk of SUDI.

### Research questions

1. How, why, for whom, to what extent and in what circumstances do targeted infant safe sleep resources (the Baby Sleep Project resources) improve the uptake of safer sleep advice?
2. What are the mechanisms by which the Baby Sleep Project resources work to improve the uptake of safer sleep advice?
3. What are the important contexts which determine whether the different mechanisms of the new resources work to improve the uptake of safer sleep advice?
4. In what circumstances are the Baby Sleep Project resources likely to be effective?

### Objectives

- Investigate the barriers and facilitators to using the resources with families using realist-informed interviews with three groups of health professionals.
- Examine how caregivers respond to the resources using realist-informed interviews with participating caregivers.
- Explore the changes in parental self-efficacy using the 13-item two-factor Uppsala Parental Self-Efficacy about Infant Sleep Instrument (UPPSEISI).^24^
- Assess adherence to infant safer sleep advice using self-reported sleep surveys
- Evaluate intervention implementation using the NoMAD Implementation measure based on Normalisation Process Theory (www.normalizationprocess.org).^25^

## Methods and analysis

### Methodology and development of the initial programme theory

Realist evaluation (RE) considers what works, for whom and under what circumstances, recognising that nothing works the same way for everyone.^26 27^ As a theory-based approach to evaluation, RE assumes that programmes do not work in the same way everywhere and for everyone, but this may be difficult to observe because the underlying mechanisms and contextual variations are rarely considered.

Interventions interact with the *context* within which different *mechanisms* can be triggered leading to *outcomes*. For our purposes, mechanisms include the cognitions and decision-making of parents and caregivers about how and where their babies will sleep. We are fortunate that a body of research exists for us to draw on, to provide evidence of the prerequisites that can support (or hinder) parents and caregivers to follow safer sleep advice.

Our initial programme theory informs the choice of methods for data collection and analysis. REs have been applied successfully to other interventions and provide potentially transferable insights by indicating theoretical domains that could be generalised to other settings.^28 29^

Realist theory development engages statements describing the context of the intervention, potential mechanisms and subsequent outcomes, together known as ‘C-M-O (context/mechanism/outcome) configurations’. By using both qualitative and quantitative methods, we hope to be able to generate data for these configurations, which relate specifically to our research questions.

The theory should include the how and the why of how the intervention delivers on the outcomes described.^30 31^ Several sources of data were used to develop the initial programme theory, describing the functions of the intervention with a focus on causal mechanisms and outcomes. The sources of data include (1) qualitative interviews with health professionals and families with infants at risk,^32^ (2) a synthesis of qualitative literature on decision-making for the infant sleep environment,^13^ (3) a systematic review of interventions to increase uptake of safer sleep advice^33^ and (4) a ‘COM-B’ (behaviour change model) analysis of this systematic review.^34^

**Figure 1:**
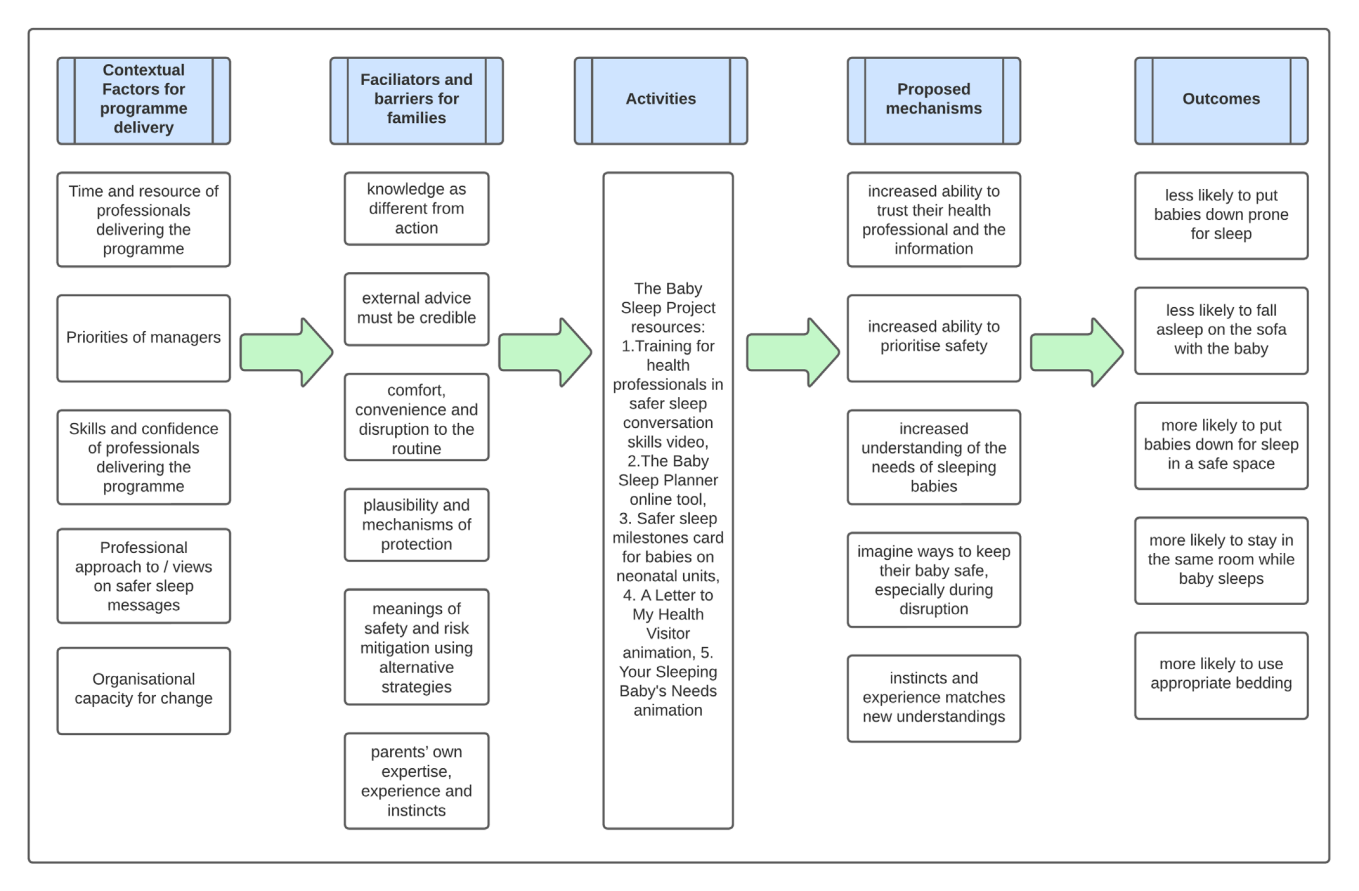
Visual representation of the initial programme theory.

Each source of data contributed to the development of configurations describing the context (C) of our intervention, potential mechanisms (M) and subsequent outcomes (O). Initial ‘C-M-O’ configuration statements were used to inform the initial theory, shown in figure 1.

Our initial programme theory states that:

- Where health professionals value the importance of conversations about safer sleep and are willing to deliver the tools in a non-judgemental, curious way, they will provide caregivers with information about their baby’s individual risk and the opportunity to plan for safety (C)
- This will increase caregiver's confidence and ability to prioritise safety, especially during busy nights, and imagine ways to provide a safer environment for their baby (M)
- Reducing their risks for SIDS by making sure their baby sleeps in a safe environment (O).

### The intervention

‘The Baby Sleep Project’ intervention comprises a suite of web-based resources for health professionals and families which aims to support safer infant sleep. The suite includes an online risk assessment and planning tool (the Baby Sleep Planner), a milestones card for use in neonatal units, two coproduced animations by young parents and a training video for health professionals.

#### The Baby Sleep Planner

The Baby Sleep Planner is a web-based tool which uses an algorithm^21^ to calculate an individual baby’s risk for SIDS and provides a planning tool for caregivers to plan for a safe sleep environment, especially during times when the normal routine is disrupted. The tool was developed as part of a National Institute for Health and Care Research (NIHR) RfPB grant (NIHR202230) via a co-design process, with associated process evaluation.^32^ The tool allows for the sleep plan to be downloaded as an image to the user’s phone for sharing with wider friends and family.

#### Safer sleep milestones card for babies on neonatal units

Babies who spend time in neonatal units are sometimes cared for in ways that do not fit the national safer sleep advice (including sometimes being placed prone in the incubator during the first weeks of life). Families reported that it can be confusing to be told to change their infant’s sleep environment once home and would have appreciated knowing why, and having this modelled while their baby was still on the unit. The safer sleep milestones card uses five milestones that can be expected to be reached while the baby is on the unit and each one can be ticked off as they are achieved. The card was developed as part of an NIHR Advanced Fellowship (NIHR300820) and via a collaboration with University Hospitals of Leicester NHS Trust neonatal team and Stork programme facilitators, charities The Lullaby Trust and Bliss. A co-design process was followed engaging caregivers of infants who had spent time on neonatal units and unit staff, consultants and the Leicester neonatal homecare team. The card is available in four languages: Polish, English, Gujarati and Urdu.

#### Coproduced animations

Two animations were coproduced in 2023 by a group of four young parents living in temporary supported housing in Bristol, UK. The first video aimed at health professionals: ‘A Letter To My Health Visitor’ shares their personal experiences of talking about safer sleep with their health professionals. The second video ‘Your Sleeping Baby’s Needs’ is for parents and explains how safer sleep messages can protect infant airways. The paper fully describing this coproduction process is in preparation for future publication.

#### Health professional training video

The training video explains the background to the project and goes through each of the available resources in the suite, explaining how they are designed to work and how to use them with families. The video also contains a chapter on ‘effective conversations with families’ which describes some of the key conversation skills that have been advocated for by recent research, discussions with families and interviews with health professionals.^32^

### Setting and context

Five sites have been recruited to the study: three neonatal units in inner city hospitals in England (Bristol, Leicester and Hull), one provider of health visiting and family nurse partnership services in four regions in England (Bath and North East Somerset, Wiltshire, Lancashire and Essex) and one NHS Trust providing health visiting and family nurse partnership services across Cheshire and Merseyside. Sites were recruited based on recent data showing regions with higher-than-average unexplained infant death rates.^1^

### Design

**Figure 2:**
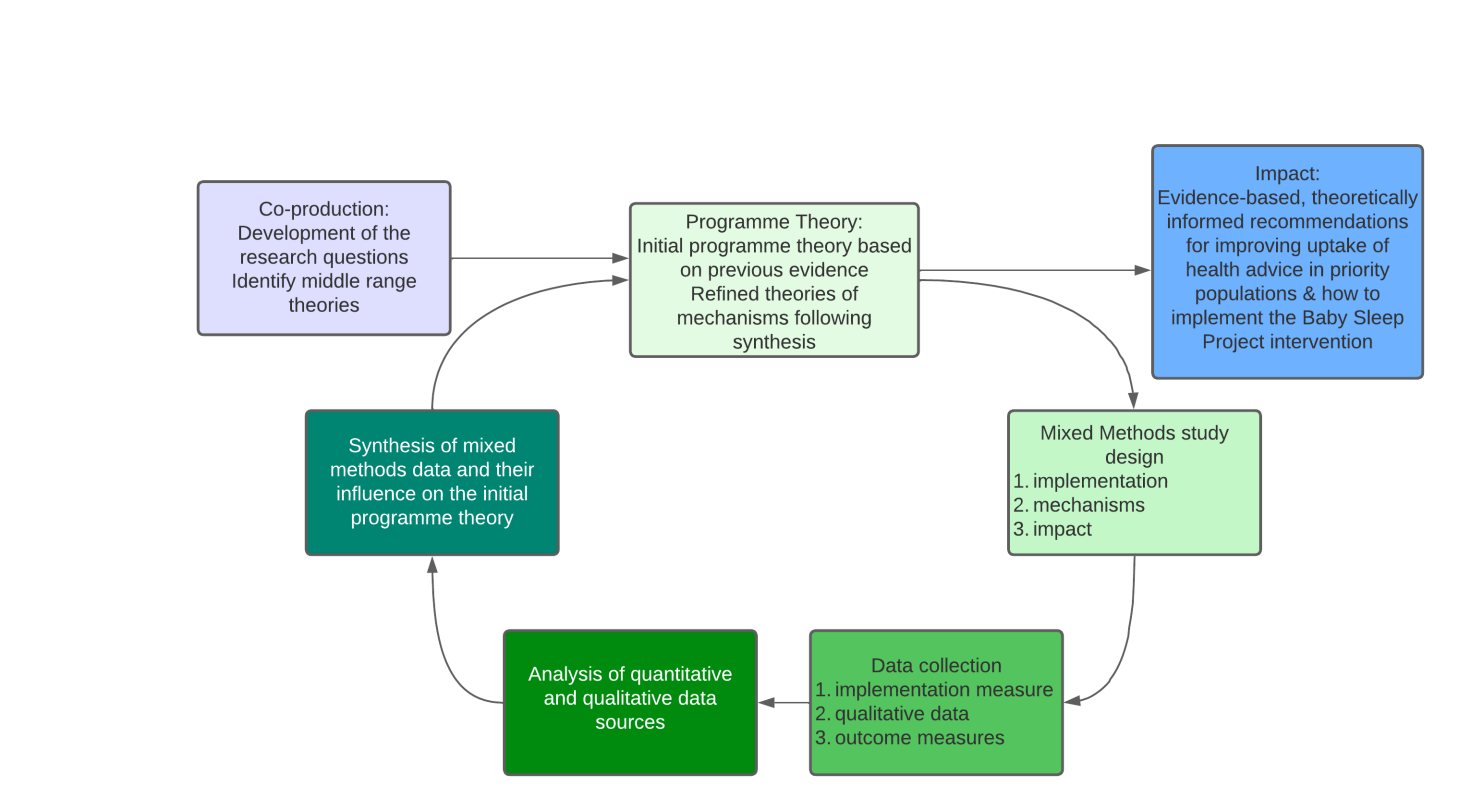
Realist evaluation cycle.

The RE study is a mixed-methods pre- and post-study design using quantitative surveys, and qualitative interviews. There will be a 3-month pretraining phase to collect baseline data with no intervention period and refine data collection methods. Figure 2 shows the realist research cycle we will engage with.

### Participants

We intend to include between 40 and 50 health professionals in total (see ‘Sample size’), spread across health visiting teams, neonatal teams and family nurse partnership teams. We are aiming to recruit 180 parents or primary caregivers of infants either still pregnant or with infants under 2 weeks old at the time of recruitment, receiving services from recruited health professionals. Parents or primary caregivers will be aged 16 or over. One parent or caregiver per child can be included.

### Recruitment

We will approach teams of health visitors, neonatal nurses and specialist nurses (family nurse partnerships) working in regions with higher-than-average rates of unexplained infant mortality and invite them to take part in our evaluation. These health professionals will be given an information sheet by the research team about the study with information about taking part. Recruitment started on 8 April 2024 and will end by 31 April 2025.

### Consent

Written consent to take part in the surveys and interviews will be obtained by members of the research team from both health professionals and parents/caregivers. All users who access the intervention will be asked for their consent for their data to be used within the study in order to improve the resources for other families. A consent statement will be displayed on the landing page before accessing the resources.

### Pre-training data collection

Health professionals who agree to take part will be asked to approach eligible family members to ask for consent to be contacted by the study team about completing two surveys about their baby’s sleep. Health professionals will show eligible family members a study details postcard, inviting them to take part in the study. For family members with literacy difficulties or where they do not speak English, study information can be read with support from the health professional, Language Line, or another family member, if appropriate. We will ask health professionals to promote the surveys with caregivers at the first point of contact in the antenatal or early postnatal period or shortly after admission to a neonatal unit. Health professionals will be asked to record that they have passed details to the study team in the family member’s medical notes. Caregivers who contact the research team or give permission for their contact details to be shared by their health professional will be contacted by a member of the team with an information sheet and link to a short video explaining the study, giving the family time to think about participation, ask questions and go through full informed consent.

### Intervention activities

When each health professional has sent us details for at least 5 caregivers or where we have reached the target of 90 caregivers completing the first survey, we will provide health professionals with full access to the new resources and training materials, with online support from the study team, via a named Baby Sleep Project Champion at each of the study sites. The training will be completed online, on a dedicated password-protected webpage, with full access to the online training video, the Baby Sleep Planner, animations, downloadable copies of the safer sleep milestones cards and downloadable flyers with QR codes for the Baby Sleep Planner and animations. We will also post hard copies of these flyers to health professionals individually on completion of the training. Health professionals will receive a certificate of completion of the training for their portfolio.

We will encourage health professionals to use the new resources primarily with caregivers who are eligible for extra support, with the same eligibility as before, though they will be different caregivers due to the baby’s age requirement. Eligible caregivers will be different for different teams, and for some it will be all their clientele, for example, family nurse partnership nurses and neonatal teams. Caregivers can see and use the resources without taking part in the study. We will ask health professionals to approach all those who have seen or used the resources to ask for consent to be contacted by the study team. We will ask health professionals to invite caregivers who have not previously seen the resources, so as not to contaminate the pretraining data collection, and we will allow time for a short gap before introducing the resources to reduce this risk. Where pretraining caregivers have completed both surveys, health professionals will be able to use the new resources with them if they wish to do so.

### Health professional research activities

We will ask all health professionals who take part in the study to complete brief implementation (NoMAD) questionnaires^25 35^ at three timepoints (immediately following the training, 2 months post-training and 4 months post-training) and ask a subset to take part in a qualitative interview. NoMAD questionnaires allow us to investigate the implementation processes and behaviours of the professionals taking part in the study, which will require collective action to become an embedded part of postnatal care.

### Caregiver research activities

**Figure 3:**
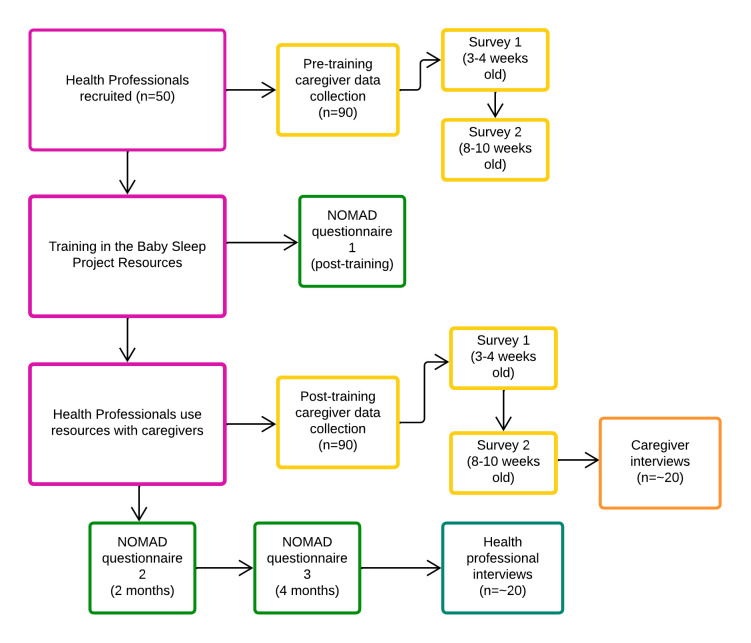
Flowchart of evaluation study.

We will invite caregivers who give consent to take part to complete online surveys when their babies are 3–4 weeks old and again when they are 8–10 weeks old (or longer where there has been a hospital stay). Two timepoints will provide evidence of any sustained effects from the intervention and covers the period of greatest risk for SIDS, according to the most recent data.^1^ We will also invite a subset to take part in a qualitative interview, pursuing a range of background characteristics relevant to SUDI risk. Figure 3 shows the intervention and research activities for health professionals and caregivers taking part in the study.

### Sample size

Using 90% power and 5% significance, proportional differences between 13% and 24% will be detected (depending on the prevalence of the variable) using 90 respondents before and 90 respondents after the study. A much larger definitive study is planned, but these findings will at least give us reasonable CIs of the differences to help generate a future sample size calculation. We aim to recruit 40–50 health professionals in total, aiming for up to 10 neonatal staff, 25 health visitors and 15 family nurse partnership nurses. Each site will have a health professional champion and a clinical principal investigator.

We will ask health professionals to approach at least five caregivers each *before* the intervention training (asking for consent to be contacted by the research team) and five each *afterwards*, assuming a 30–40% sign-up rate of approximately 3–4 caregivers per health professional in total. We should be able to recruit approximately 90 caregivers before and 90 caregivers after the intervention, total number of caregivers approximately n=180. We expect the minimal loss to follow-up due to the voucher incentive for the second survey and will over-recruit slightly on the first survey (up to 95) to minimise this risk. Due to the long recruitment period, we will have time to monitor follow-up rates and adjust accordingly, if required.

### Data collection

#### NoMAD questionnaires (post-training)

NoMAD questionnaires will be completed by participating health professionals at three timepoints (post-training, 2 months and 4 months) and will be used to assess implementation across each of the four domains of Normalisation Process Theory (coherence, cognitive participation, collective action and reflexive monitoring).^25^ These questionnaires will be analysed to look for differences between roles, regions and changes across each timepoint. Petal charts will allow us to visualise these findings.

#### Parental surveys (pre- and post-training)

Online self-efficacy measures and sleep surveys will be sent to caregivers at 3–4 weeks after birth and 8–10 weeks after birth (or longer where there has been a hospital stay). Surveys will be completed online (using JISC online survey platform), but can also be completed over the phone with a member of the research team or via an online interpretation service. We will also collect data on maternal age, parity, smoking/vaping during pregnancy, NICU admission, baby’s sex, birth weight, partner support, partner smoking status, usual infant sleep location/position and sleep location/position last night (reflecting the night immediately before the survey). The 13-item two-factor UPPSEISI will be used to measure parental self-efficacy. Our outcome measures will be the prevalence of non-supine sleeping positions and hazardous co-sleeping (co-sleeping with an infant on a sofa or in a bed with an adult who has consumed alcohol, smokes, has taken drugs that may make them drowsy or with an infant that was born before 37 weeks gestation or under 2500 g birth weight). A copy of the survey can be found in online supplemental file A. Survey responses to our main outcome data will be mandatory within the survey software.

#### Qualitative interviews (post-training)

Interviews with health professionals will take place 8–12 weeks after training in the intervention to give them time to have had experience with it with several families. Topic guides will be informed by our C-M-O configurations and initial programme theory (see online supplemental file B).

Caregivers in the intervention group will be invited to a semistructured interview via consent to be contacted collected in the survey. The topic guide will be informed by our C-M-O configurations and initial programme theory (see online supplemental file C).

### Integration of data sources and analysis

Data sources will be used to develop the programme theory by comparing and contrasting it with our initial programme theory. Data will be used to support, refine or refute this initial theory. We will begin by using our initial theory as a coding framework for the qualitative interviews (parent and professionals) analysis. This analysis will be done in NVivo using both inductive and deductive coding to allow for the identification of new conceptual themes not present in our initial programme theory. These themes will be discussed with the research team to develop C-M-O configurations. We will then use the quantitative data sources (NoMAD and parental surveys) to look for how they do or do not support these configurations. Parental surveys will be downloaded and anonymised and analysed to investigate differences in infant care practices relating to the intervention between pre- and post-training. We will investigate whether hazardous co-sleeping or non-supine sleeping as a usual practice or ‘last night’ changes between the two groups. These distinct measures will be documented separately. We will also compare parental self-efficacy between the two groups. As these surveys will be completed by caregivers of different babies, we will look closely at any differences in the characteristics of these individuals. Any characteristics that significantly differ between the groups will be adjusted for in the analysis. Data will be analysed using STATA to compare responses before and after the intervention is introduced, and proportional differences in outcome measures between 4 and 8 weeks. We will also compare subgroups of responses based on lower (<115) and higher (115+) risk scores using our recently developed risk scoring algorithm.^21^ Quantitative data will be compared within and between sites and between lower-risk and higher-risk participants to explore differences and inform the development of the C-M-O configurations. Normalisation Process Theory and the COM-B model will support cross-case comparisons of our C-M-O configurations to support the iterative development of the programme theory alongside the integration of data sources. The final programme theory will be presented in a diagram with a corresponding descriptive account. All data will be held on University of Bristol servers with access only available to the research team and analysed using STATA statistical software.^36^

#### Incentives to participate in the study

Parents/caregivers who complete the online measures at both timepoints will be offered a £10 voucher. Those who also take part in an interview will be offered a £20 voucher to thank them for their participation.

### Patient and public involvement

We have continuously sought caregiver views on our study methodology via attendance at groups run via local children’s centres and through our network of local health visiting teams. Comments from parents at parent groups and regular events specifically for families highlight how much pressure is often on mothers as primary carers to keep to ‘safety rules’ and suggest that involving partners, peers and family members who are both influential in sharing advice and looking after the baby would support any future efforts to decrease risks. Parents also told us that resources they can use need to be online and preferably not in an app.

In addition, our parental advisory group consists of 15 parents with young babies. They met to discuss this protocol in its draft form, and we have asked them specific questions about recruitment and data collection. The group meets with a quorum of two at any meeting and email communication supplements each meeting. The group suggested ways to maximise recruitment of other family members, including having a range of incentives to offer and reminders to those who agree to be contacted. They have also pilot-tested our caregiver surveys and commented on study information sheets. These suggestions have been incorporated into the current protocol. The group will continue to meet regularly to provide advice and support on good ways to encourage parents/carers to take part in surveys and interviews, interpretation of findings and dissemination to wider audiences. Group members are supported by the inclusion of an induction into the role of advisory group member where we have agreed ‘safety ground rules’ for our meetings. UK Standards for Public Involvement, as recommended by the NIHR,^37^ are used to provide support with voicing opinions, understanding research terms, signposting for emotional support and ensuring barriers to accessing meetings are broken down and that vouchers/payments for participation are paid promptly.

### Study oversight

Our professional advisory steering committee for the study comprises a neonatal consultant, a professor of anthropology and infant sleep, the CEO of a national safer sleep charity, a professor of nursing, midwifery and infant care, a specialist health visitor for Gypsy, Roma and Traveller families and a representative of the local Integrated Care Board. The steering committee meets twice yearly to provide overall supervision of the study on behalf of the NIHR and sponsor. The committee will concentrate on the progress of the study, protocol adherence, interpretation and dissemination of the findings.

### Withdrawal or discontinuation from the study

Participants will have the right to withdraw from the study at any stage. They will be given the option to withdraw all their data, or to discontinue but allow data already collected to be used.

### End of the study

The study will end for health professionals when they have completed NoMAD questionnaires at three timepoints, and for a subgroup, when they have completed an interview. The study will end for parents/caregivers when they have completed their online sleep and self-efficacy measures, and for a subgroup, when they have completed an interview.

## Ethics and dissemination

### Assessment and management of risk

The main ethical issue with this study is the sensitivity around discussing sudden and unexpected death in infancy. If carers appear to be upset during an interview, we will use a checklist to make sure that we take the right steps, document the event thoroughly and follow up or report where required. Each family member who takes part in an interview will be sent a copy of a list of support organisations they can contact for advice about safer sleep for babies and for support with mental well-being during parenthood. This will be emailed by the research team, and a paper copy offered to be sent in the post. It is unlikely, but possible, that the interviews may reveal issues of safeguarding for infants, children and young people. If necessary, relevant organisations will be consulted, including the National Society for the Prevention of Cruelty to Children or local children’s care services. We would make a formal referral when required to a statutory child protection agency or the police. This is in line with statutory requirements that apply to all professionals, whether or not they are involved in research. Risks to the research team will be managed by adherence to the University’s ‘Health and safety guidance for research undertaken in the community’ policy document.^38^

### Dissemination

The data arising from the study will be owned by the University of Bristol. On completion of the study, a full study report will be prepared along with papers for publication detailing the results from the evaluation. The RAMESES reporting standards will be used to provide a comprehensive report on the evaluation.^39^ All qualitative sampling, data collection, analysis and reporting will also comply with the consolidated criteria for reporting qualitative research (COREQ) checklist.^40^

With guidance from our family and professional advisory groups, we will aim to disseminate the findings via peer-reviewed publication in relevant academic journals within 12 months of the end of the study. Participants will be notified of the findings of the study with a confidential newsletter with a plain English summary of the overall findings, with links to open-access publications.

## supplementary material

10.1136/bmjopen-2024-091414 online supplemental file 1

10.1136/bmjopen-2024-091414 online supplemental file 2

10.1136/bmjopen-2024-091414 online supplemental file 3
