# Supplementary material for: Baby Sleep Project Protocol: a realist evaluation of an intervention to reduce preventable infant mortality
Source: BMJ Open. 2025 Feb 13;15(2):e091414. doi: 10.1136/bmjopen-2024-091414 (PMC11831312; doi:10.1136/bmjopen-2024-091414)
Supplement: online supplemental file 1 [file bmjopen-15-2-s001.pdf]

The Baby Sleep Project Survey

Page 1: About the Baby Sleep Project

About the Baby Sleep Project

If you are over 16 and a parent, carer or someone who looks after a baby up to 12 months old we'd like to invite you to take part in this online survey. The survey is about how, and where babies sleep, related to the advice you might have heard to do with cot death, or Sudden Infant Death Syndrome (SIDS).

The Baby Sleep Project will use the information from the survey to design better ways to support families with sleep in the first few months of life. We'd love to hear from anyone who looks after a young baby especially young people (16-25) and people who are not the mother, e.g. Grandparent, father, partner, or other family member. We'd like to hear from younger caregivers and wider family and friends because their views are important and not usually asked for in research like this.

The study is being run by researchers at the University of Bristol – Dr Anna Pease and Dr Becky Ali. It is funded by the National Institute for Health Research and has been approved by the Health Sciences Faculty Research Committee at the University of Bristol. You can find out more about the study here: www.babysleepresearch.co.uk or by emailing babysleep@bristol.ac.uk

Please go to the next page to find out more and take part in the survey.

Page 2: More Information

More Information

This survey is about where and how your baby (or the baby you look after) sleeps. It includes questions about you and your baby and your family circumstances. It asks about what usually happens with sleep and about what happened last night.

If you are looking after twins or more than one baby please fill in the survey for one baby at a time.

We do not collect any names, or addresses – there is an option at the end to enter your mobile phone number or email if you would like to be sent the results, or if you consent to being contacted about future research, but this is optional. All your answers on the survey will be stored separately to any contact details you enter, so we will not be able to link survey data with any personal details.

We will keep the data secure by storing it on University of Bristol computers, with access only available to our research team. At the end of the study an anonymised dataset with only study numbers as identifiers will be stored on the University of Bristol data repository (https://data.bris.ac.uk/data/). Access may be granted to future researchers if they have legitimate scientific requests and all ethical approvals are met. Please see our full Data Privacy Notice for further details.

You don't have to do the survey all in one go, you can click 'finish later' and come back to it.

All the questions are confidential – we hope you can be honest as this helps us greatly in the research.

Once you have submitted your answers you cannot withdraw them again, but if you change your mind about taking part you can close your browser and choose not to continue.

At the end of the survey you have the option of entering your email address if you would like to enter a prize draw to win one of three £50 shopping vouchers, and/or if you consent to being contacted about an interview on the same topic as the survey, and/or if you would like to be sent a copy of the results of the survey. Each choice is optional and you can submit the survey without entering an email address.

Page 3: Eligibility check

To be able to take part in this survey, you must be aged 16 or older, looking after a baby younger than 12 months old and live in England. Please confirm below: ✱ *Required*

- Yes, this is me
- No, this is not me

Would you like to continue to the survey?

- Yes
- No

Page 4: Information about you

Are you the baby's main carer? ✱ *Required*

- Yes, I'm the main carer
- No, I support the main carer

How many children have you had in total?

- 1
- 2 or 3
- 4 or more

What is your relationship to the baby? (e.g. mother, father, step-dad, aunt, family friend) ✱ *Required*

- Mother (biological)
- Father (biological)
- Mother’s partner (or non bio father/mother or step father/mother)
- Adoptive mother
- Adoptive father
- Aunt
- Uncle
- Family friend
- Neighbour
- Grandmother
- Grandfather
- Step grandmother
- Step grandfather
- Foster mother

- Foster father
- Great uncle
- Great aunt
- Other
- Sibling (brother or sister)

If you selected Other, please specify:

Are you supported by a partner?

- Yes
- No

Does your partner smoke tobacco?

- Yes
- No

Page 5: Information about baby

What is the baby's sex? ✱ *Required*

- Male
- Female
- Other (Intersex)

How old is the baby in weeks? ✱ *Required*

- 0-4 weeks (0 months)
- 5-8 weeks (1 month)
- 9-12 weeks (2 months)
- 13-16 weeks (3 months)
- 17-20 weeks (4 months)
- 21-24 weeks (5 months)
- 25-28 weeks (6 months)
- 29-32 weeks (7 months)
- 33-36 weeks (8 months)
- 37-40 weeks (9 months)
- 41-44 weeks (10 months)
- 45-48 weeks (11 months)
- 49-52 weeks (12 months)

How old is the mother (or the person who gave birth)? ✱ *Required*

- Under 21 years
- 21 - 24 years
- 25 or over

What option fits the baby's ethnicity best?

If you selected Other, please specify:

Is the baby living with you?

- Yes
- No

Page 6: Further information about baby

What was the baby's birthweight? ✱ *Required*

- Under 1750g (3lbs 13ozs)
- Between 1750g (3lbs 13ozs) and 2499g (5lbs 8ozs)
- Over 2500g (5lbs 8ozs)
- I don't know

Did the baby spend any time in the Neonatal Intensive Care Unit (NICU) when they were born? ✱ *Required*

- Yes
- No
- I don't know

Was the baby born before 37 weeks gestation (were they ‘preterm’)?

- Yes
- No
- I don't know

Is the baby a twin or a triplet?

- Yes, a twin
- Yes, a triplet
- No, just one baby

- Other

If you selected Other, please specify:

How is the baby being fed currently?

- Any Breastmilk (either exclusively, with formula or solids)
- No Breastmilk (formula or on solids only)
- I don't know

Page 7: Baby's sleeping arrangements

Please select all the surfaces on which the baby **has ever slept on/in.** If you're not the baby's main carer just select the ones you know about:

- ☐ Cot/moses basket/crib
- ☐ Adult bed alone
- ☐ Adult bed with sleeping adult
- ☐ Sofa alone
- ☐ Sofa with sleeping adult (ever)
- ☐ Co-bedding with sibling/s in a cot/moses basket/crib (twins or multiples only)
- ☐ Car seat
- ☐ Sling
- ☐ Buggy/pushchair
- ☐ Bouncy chair
- ☐ Other

If you selected Other, please specify (you can add more than one place):

Which is the **most common** place for the baby to sleep **during the day**? You can select more than one answer: ✱ *Required*

- ☐ Cot/moses basket/crib
- ☐ Adult bed alone
- ☐ Adult bed with sleeping adult
- ☐ Sofa alone
- ☐ Sofa with sleeping adult (ever)
- ☐ Co-bedding with sibling/s in a cot/moses basket/crib (twins or multiples only)

- [ ] Car seat
- [ ] Sling
- [ ] Buggy/pushchair
- [ ] Bouncy chair
- [ ] Other

If you selected Other, please specify:

Which is the **most common** place for the baby to sleep **during the night**? You can select more than one answer: ✱ *Required*

- [ ] Cot/moses basket/crib
- [ ] Adult bed alone
- [ ] Adult bed with sleeping adult
- [ ] Sofa alone
- [ ] Sofa with sleeping adult (ever)
- [ ] Co-bedding with sibling/s in a cot/moses basket/crib (twins or multiples only)
- [ ] Car seat
- [ ] Sling
- [ ] Buggy/pushchair
- [ ] Bouncy chair
- [ ] Other

If you selected Other, please specify:

What position is the baby **usually** put down for sleep in **during the day**? ✱ *Required*

- On their back
- On their front
- On their side
- I don't know
- I can't remember
- Other

If you selected Other, please specify:

What position is the baby **usually** put down for sleep in **during the night**? ✱ *Required*

- On their back
- On their front
- On their side
- I don't know
- I can't remember
- Other

If you selected Other, please specify:

Has baby ever been found sleeping with blankets or any other soft material covering their face?

- Yes
- No
- I don't know
- I can't remember

Thinking about last night (from baby’s bedtime to getting up in the morning), where did the baby sleep **last night**? You can select more than one answer: ✱ *Required*

- Cot/moses basket/crib in same room as sleeping adult
- Cot/moses basket/crib in own room
- Adult bed alone
- Adult bed with sleeping adult
- Co-bedding with sibling/s in a cot/moses basket/crib (twins or multiples only)
- Sofa alone
- Sofa with sleeping adult
- Other

If you selected Other, please specify:

Page 8: Further questions about baby's sleeping arrangements

Thinking about last night (from baby’s bedtime to getting up in the morning), was anybody co-sleeping with the baby at any point last night? (Co-sleeping means an adult and a baby both asleep on the same surface, for example on a bed or a sofa) ✱ *Required*

- Yes
- No
- I don't know
- I can't remember

Who was co-sleeping with the baby?

- Me
- Me and my partner
- Other

If you selected Other, please specify:

Where was this?

- Adult bed
- Sofa
- Chair
- Other

If you selected Other, please specify:

Was co-sleeping done on purpose (you/they planned to fall asleep this way)?

- Yes
- No

What was the reason for co-sleeping with the baby? (Please select all that apply)

- Usual practice
- Baby unsettled
- Staying overnight away from home
- No baby bed available
- Make it easier to breastfeed
- To get more sleep
- Baby unwell
- Other

If you selected Other, please specify:

Were there any objects in the co-sleeping space last night?

- Yes
- No

- I don't know
- I can't remember

Were these objects...(select all that apply)

- Soft toys
- Duvet
- Pillow
- Sleep pod/Nest
- Sleep positioner
- Other

If you selected Other, please specify:

During baby's sleep **last night** were any of the sleeping surfaces tilted? ✱ *Required*

- Yes
- No
- I don't know
- I can't remember

If yes, why were they tilted?

- Reflux
- Other

If you selected Other, please specify:

If baby spent any time sleeping in their own bed (the cot/moses basket etc.) last night, were there any objects in the bed with them? ✱ *Required*

- Yes
- No
- I don't know
- I can't remember

What were those objects? (select all that apply)

- Soft toys
- Bumper
- Pillow
- Sleep pod/Nest
- Sleep positioner
- Other

If you selected Other, please specify:

What was the reason for the object/s being there?

Page 9: Last night's sleep

What position was the baby **put down** to sleep last night? ✱ *Required*

- On their back
- On their front
- On their side
- I can't remember
- I don't know
- Other

If you selected Other, please specify:

Is this how you usually put them down for sleep?

- Yes
- No

What position was the baby lying in when getting up in the morning?

- On their back
- On their front
- On their side
- I don't know
- I can't remember
- Other

If you selected Other, please specify:

What clothing was the baby wearing for sleep? (select all that apply)

- [ ] Vest
- [ ] Babygrow
- [ ] Sleeping bag
- [ ] Togged sleeping suit
- [ ] Hat
- [ ] I don't know
- [ ] I can't remember
- [ ] Other

If you selected Other, please specify:

What bedding was over the baby **when put down for** sleep last night? ✱ *Required*

- [ ] Sleeping bag or togged sleep suit
- [ ] Blankets
- [ ] Other

Was the blanket tucked in at the sides of the mattress or left loose?

- Tucked in at the sides
- Left loose

If you selected Other, please specify:

Was the baby given a dummy for sleep at all last night?

- Yes
- No
- I don't know
- I can't remember

Was the dummy in the baby’s mouth when the baby woke up?

- Yes
- No
- I don't know
- I can't remember

Page 10: Changes to the usual routine

Were there any changes to the usual routine last night? E.g. feeding routine, sleeping routine, visiting or having visitors, change in carers or caring practice, sleeping position, dummy use, carers having a drink ✱ *Required*

- Yes
- No
- I don't know
- I can't remember

If yes, please choose the one(s) that best describe the reasons for this change (you can pick more than one)

- Baby fed less than usual
- Baby was unwell
- Baby woke more often than usual
- Stayed overnight away from home
- Had visitors at home in the evening or overnight
- Change in dummy use
- Main carers had a drink
- Someone else was looking after the baby
- Baby was put down for sleep in a different position
- Other

If you selected Other, please specify:

These next questions ask about drink and drug use. This information is important to our

research so we hope that you can answer honestly. We won't be able to identify you from your answers.

Do you smoke tobacco? ✱ *Required*

- Yes
- No

Did you (or the person who gave birth) smoke tobacco during their pregnancy? ✱ *Required*

- Yes
- No
- I don't know

In the last 24 hours, did any of the **main carers** (whoever was looking after the baby last night) have any alcohol? ✱ *Required*

- Yes
- No
- I don't know

If yes, was this more than 2 units (one standard measure of spirits, one small glass of wine, one small can of beer)?

- Yes
- No

In the last 24 hours, did any of the main carers (whoever was looking after the baby last night) have any recreational or prescription drugs? ✱ *Required*

- Yes
- No
- I don't know

Were these recreational drugs? (e.g. cannabis, cocaine, heroin)

- Yes
- No
- I'd rather not say

What is the name of these drugs?

Were these prescription drugs? (e.g. any medicine prescribed by a doctor or clinician)

- Yes
- No
- I'd rather not say

What is the name of these drugs?

Page 11: A final few questions

Has this baby had any involvement from social workers? ✱ *Required*

- ◯ Yes
- ◯ No
- ◯ I don't know

Did you have a conversation with a health professional about SIDS or cot death risks? ✱ *Required*

- ◯ Yes
- ◯ No
- ◯ I can't remember

Do you remember which topics they mentioned? Select all that apply

- ☐ Back sleeping
- ☐ Advice about Bedsharing
- ☐ Not smoking
- ☐ Feet to foot
- ☐ Not Sofa sharing
- ☐ Head covering
- ☐ Advice about safer sleep for twins or multiples
- ☐ Other

If you selected Other, please specify:

The following questions help us to know more about who is answering this survey and we won't be able to identify you from the answers you give.

What level of education do you have?

- ○ Below GCSE level
- ○ GCSE level or above

Is your total household income ABOVE £17,940 per year?

- ○ Yes
- ○ No

There is another part to this study where we would like to interview some people who have completed this survey. We'd like to know more about your baby's sleep and you. Everyone who takes part in an interview will be offered £20 to say thank you. Please let us know below if you would be happy to hear more information about this - taking part is up to you and you don't have to decide until after we have contacted you.

Would you be happy for us to contact you only for the following reasons? (select all that apply) ✱ *Required*

- ☐ Yes, to hear about a possible interview
- ☐ Yes, to be sent a summary of the results
- ☐ Yes, to be entered into the prize draw to win one of 3 x £50 vouchers
- ☐ No thanks

Thank you for agreeing to let us contact you. Please provide your email address (we can't enter you into the draw without this):

Please enter a valid email address.

How did you hear about this survey? ✱ *Required*

- From my health visitor/support worker
- Poster at children's/health centre
- Facebook advert
- Facebook group post
- Bliss
- Instagram
- A friend of family member told me
- Through the website (babysleepresearch.co.uk)
- Other

If you selected Other, please specify:

Page 12: Final page

Thank you for taking the time to complete our survey, your responses are very helpful and important for our research.

Please share this survey with others who may wish to take part: https://sps.onlinesurveys.ac.uk/baby-sleep-project

If you would like to get in touch about this study our email is babysleep@bristol.ac.uk

You can also find out more information about this and other Baby Sleep research projects on our website: www.babysleepresearch.co.uk

---

Key for selection options

4.b - What option fits the baby's ethnicity best?

- White: English, Welsh, Scottish, Northern Irish or British
- Irish
- Gypsy or Irish Traveller
- Any other White background
- White and Black Caribbean
- White and Black African
- White and Asian
- Asian British (mixed ethnicity)
- Indian
- Pakistani
- Bangladeshi
- Chinese
- Any other Asian background
- Black British
- African
- Caribbean
- Any other Black, African or Caribbean background
- Arab
- Any other ethnic group
