# Supplementary material for: Baby Sleep Project Protocol: a realist evaluation of an intervention to reduce preventable infant mortality
Source: BMJ Open. 2025 Feb 13;15(2):e091414. doi: 10.1136/bmjopen-2024-091414 (PMC11831312; doi:10.1136/bmjopen-2024-091414)
Supplement: online supplemental file 2 [file bmjopen-15-2-s002.pdf]

The Baby Sleep Project - Semi-structured Interviews with Health Professionals

Introduction

- Thank participant for meeting
- Check participant is still happy to be involved with the research
- Explain recording device / confidentiality
- Explain purpose of the interview and that participant does not have to talk about anything they feel uncomfortable with and can stop at any time for any reason
- Check consent form is signed
- Record verbal consent on separate audio file if necessary

Interview Part 1: Knowledge and understanding of SIDS

- Prior to taking part in the Baby Sleep Project, what kind of training did you receive about SUDI?

Interview Part 2: The resources

- What were your thoughts on the Baby Sleep Project training video?
- What did the training/resources provide that was new? (probe for greater depth to understand mechanism rather than activity provided e.g. Skills attitude change rather than 'training')
- Which of the Baby Sleep resources have you been using with families you support?
- Can you describe your experience of using each of the resources?
    - How did you feel when you were using that resource?
    - What would you have done differently before you had this resource?
- Do you know if any families have made any changes to how they put their baby down for sleep as a result of seeing the resources?
- When you shared the resources with families, did any of them comment on the information provided – was any of the information new to them?

Interview Part 3: New Programme Theory CMOCs

- What do you consider the outcomes of the baby sleep project to have been for you?
    - Can you give an example of [outcome named by them in previous question]?
- What exactly was it about the [training/resource] that helped to cause that [outcome]?
- The training video discusses reframing conversations about sleep safety. Since watching it, have you had any thoughts about how you approach this topic with families?
- Some health professionals have told us they feel 'backed up' by the resources when sharing sleep safety advice with families – would you say this has been an outcome for you? Can you elaborate?
- What do you consider the outcomes of the baby sleep project have been for **the families** that you work with?
    - Can you give an example of that [outcome named by them in previous question]?
    - What was it about the resource they used which led to this outcome, do you think?
- We are curious about how the baby sleep project resources cause [outcomes previously identified], how do you think the programme has caused or helped to cause [outcome previously identified]?

- Some other health professionals have told us that, because the resources include information on reframing the risk advice, they found it easier to have sleep safety conversations with parents, which led to solution-focused conversations – would you say that has been your experience?
- Along with other health professionals, you are helping us to test out our ideas. One idea is that by including personalised SIDS risk assessments in the resources, parents may feel better informed of their baby's risk status and therefore more likely to engage with sleep safety practices – what do you think of this idea?
- We were worried about parents disengaging from the resource if it told them their baby was at increased risk of SIDS – has this been your experience/what are your thoughts?
- There are lots of ideas about how the baby sleep project actually works, and we think it probably works differently in different places or for different people. One of those ideas is that by [complete with mechanism, e.g. Building relationships between families and HCPs families will trust the information they are given]. Has it worked at all like that here/for you?
    - Can you give an example?
- Do you think the resources would be as/more effective if used antenatally?
  - Did you feel that families were open to discussing safe sleep with you?
  - Did you feel that the resources could be incorporated into your work easily?
    - Did you have time to discuss or use the tool with families you are working with?
    - Probe – was there any extra time allocated? Was your manager supportive?
- We created these resources with health professionals in mind, so that sleep safety conversations could be shorter and more effective. However, some health professionals told us that they simply did not have time to use any of the resources with families – what are your thoughts on this?
- If you could change something about the resources in the baby sleep project to make them work more effectively, what would you change and why?

Interview Part 4: Future directions and suggestions for improvement

- Prompt: show the health professional any resources they're not yet familiar with.
- Do you think that you will continue using the resources in your work with families?
- Do you have any suggestions for improvements in the resources?
- What was your experience of the logistics of using the resources?
    - Did your devices have enough internet access/battery life to be used properly?
- We were worried that families would not engage if health professionals 'just' gave them a QR code card and left them to it – what are your thoughts on this?

Final thoughts

- Before we finish the interview, is there anything else you'd like to add about the baby sleep project resources?
- Thank participant for their time and check details of how to pay their expenses/voucher / share study findings with them (if requested).
