# Supplementary material for: Baby Sleep Project Protocol: a realist evaluation of an intervention to reduce preventable infant mortality
Source: BMJ Open. 2025 Feb 13;15(2):e091414. doi: 10.1136/bmjopen-2024-091414 (PMC11831312; doi:10.1136/bmjopen-2024-091414)
Supplement: online supplemental file 3 [file bmjopen-15-2-s003.pdf]

The Baby Sleep Project

Topic guide for semi-structured interviews with family members

Introduction

- Thank participant for meeting
- Check participant is still happy to be involved with the research
- Explain recording device / confidentiality
- Explain purpose of the interview and that participant does not have to talk about anything they feel uncomfortable with and can stop at any time for any reason
- Check consent form is signed
- Record verbal consent on separate file if necessary

Interview Part 1:

- Can you tell me about your family? How old is your baby?
  - Do you have any other children?
- How have you been coping with your baby's sleep?
  - What have you found most challenging when it comes to your baby's sleep?

Interview Part 2: The Baby Sleep Project Resources

- We wanted to talk to you today because you received [the baby sleep project resources] from your [HCP]
  - Can you tell me what your contact with [the baby sleep project resources] has been?
  - What kind of resources did [HCP] use/share with you? (prompt by sharing an image of the BSP resources)/have you seen any of these before?
  - Did you use these resources?
    - How did you use these resources?
    - Did you find the resources useful to help you to keep your baby safe at night?
- [Go through resources used with participant] What were your initial thoughts about [resource]?
  - Did your HCP talk you through [the resource] or did use it by yourself?
  - What did you think of [aspect of the resource]? Probe about each part of the resource(s) and get participant to talk through each part of it
- What has changed as a result of seeing the resources?
  - What has changed for you?
  - What has changed for your family?
- What changed for you after seeing the baby sleep project resources?
  - Can you give an example of [a change]?
- Do you think the changes have been the same for all families? Can you imagine families that may have responded differently to the materials?
- Did you share the resources with other people who were caring for the baby? (eg. Grandma, dad, friends etc)
  - What kind of feedback did you get from them?

- We are curious about how the BSP resources might work for families. How do you think the BSP resources changed things for you? [outcome identified previously by respondent]?
    - Probe - what did the resources provide that were new (eg. Skills, attitude change etc)?
- Have the BSP resources changed the way you think or feel about [keeping your baby safe] in any way?
- What kind of conversations did your health care professional have with you about your baby’s sleep?
    - Did they talk about how to keep your baby safe?
- How would you describe your relationship with [HCP]?
    - Do you feel like you can be open with them about how you were coping?
    - Did you feel like you could be honest about how or where your baby was sleeping?
- We’ve seen that the BSP works differently for different people, who do you think it would/wouldn’t work for?

[If ppt has had previous children]

- How has your experience of sleep been different/the same with this child as it was with your previous child?
    - What did you do differently?
    - Probe – Why did you do x differently?
- If you could change something about the baby sleep project to make it work more effectively with families like yours, what would you change and why?
- What do you think we need to know, to really understand how the BSP has worked?

Final thoughts

- Before we finish the interview, is there anything else you’d like to add about the baby sleep project resources?
- Thank participant for their time and check details of how to pay their expenses/voucher / share study findings with them (if requested).
