# Supplementary material for: Baby Sleep Project Protocol: a realist evaluation of an intervention to reduce preventable infant mortality
Source: BMJ Open. 2025 Feb 13;15(2):e091414. doi: 10.1136/bmjopen-2024-091414 (PMC11831312; doi:10.1136/bmjopen-2024-091414)
Supplement: Supplementary file 4 [file bmjopen-2024-091414.reviewer_comments.pdf]

PEER REVIEW HISTORY

BMJ Open publishes all reviews undertaken for accepted manuscripts. Reviewers are asked to complete a checklist review form and are provided with free text boxes to elaborate on their assessment. These free text comments are reproduced below.

ARTICLE DETAILS

Title (Provisional)

The Baby Sleep Project Protocol: A realist evaluation of an intervention to reduce preventable infant mortality.

Authors

Pease, Anna; Lambert, Becky; Ingram, Jenny; Bradley, N; Fleming, Peter; Blair, Peter; Farr, Michelle

---

VERSION 1 - REVIEW

**Reviewer**	**1**
**Name**	**Rajai, Azita**
**Affiliation**	**University of Manchester, Faculty of Biology, Medicine and Health.**
**Date**	**25-Nov-2024**
**COI**	**I have no competing interest**

The study demonstrates a well-researched background and benefits from an experienced research team. I believe the qualitative methodology is adequately described.

Sample size calculation is well described. The estimated sample size ranges from 120-200 participants, with the final number depending on healthcare professional recruitment and response rates. While 180 approaches the upper limit, you should anticipate achieving approximately 160 participants, which remains statistically adequate. There are some points with regard to the quantitative part that requires more details.

Could you clarify the distribution plan for the baby sleep resources? Will these materials be provided to all participating families, both before and after the intervention period.

The quantitative data collection methodology requires further detail, particularly regarding the timing of measurements at 3-4 weeks and 8-10 weeks postpartum. Please specify:

- Whether responses should reflect the day immediately before the survey or participants should provide an average from the previous week etc.

- How should instances of non-supine or hazardous sleeping positions be recorded? Should the data reflect an average over the week prior to the survey? Will the two outcomes—non-

supine sleeping and hazardous sleeping—be documented separately or combined into a single measure?

- What methods will be implemented to ensure baseline characteristics are comparable between intervention and control groups so that any observed differences in outcomes can be attributed to the intervention.

- How will the missing /unanswered/survey non-responses be handled. Has the non-response/lost to follow up been considered in the sample size?

- There is no specific detail provided on how data will be recorded. Will the data/some of the data initially be documented in case notes and later transferred to an electronic system? What is the established procedure for ensuring data anonymization, as well as maintaining its quality and integrity throughout the process?

**Reviewer**	**2**
**Name**	**Ramakrishnan , Rema**
**Affiliation**	**George Institute Oxford**
**Date**	**25-Nov-2024**
**COI**	**I have no conflict of interest to declare.**

The evaluation of an intervention for infant safe sleep is important. The proposed study by Pease et al involves intervention both at the level of healthcare professionals and parents/caregivers. The intervention includes web-based resources for health professionals and families to support infant safe sleep – The Sleep Planner, Safer sleep milestones card for babies on neonatal units and videos for caregivers and training video for healthcare professionals. However, the protocol description would benefit from some clarifications and additions described below.

SPECIFIC COMMENTS

1. It is appropriate that the sample size for the qualitative aspect of the study is based on pragmatic and feasible reasons. However, unless this is an exploratory/pilot study it is not appropriate to not calculate sample size for the quantitative aspect of the study. The sample size may not be adequate for the self-efficacy measure. Since the study is in-progress, could you please report the power calculation for comparison of responses before and after the intervention, and proportional differences in outcome measures between 4 and 8 weeks and also for the subgroup analyses and for comparison between sites?

2. Please add details about how health care professionals' data from NoMAD questionnaires will be analysed. Besides this, please add information about how the analysis will account for within-site clustering effect. That is, please specify what method will be used to account for

within-site clustering when reporting proportional differences in outcome measures between 4 and 8 weeks and for the subgroup analysis by risk score categories.

3. The pre-intervention activities seem to be unclear. Page 7- lines 16-17: Please clarify what pre-training data collection includes and please clarify when the training will take place with respect to recruitment.

4. Please clarify why data will be collected at two time-points for caregivers. What is expected to change during this period?

5. Please clarify what will be done with the data from caregivers collected during the pre-training period. Presumably, these are not the same caregivers after the training. Therefore, it would be important to compare the characteristics of these and if they differed that could account for the pre-post difference in intervention on the outcome measure instead of the intervention itself.

6. The study is in-progress. Therefore, please describe the progress made other than recruitment. For example, it seems that the NoMAD questionnaire should have been completed by those recruited so far. Please provide details of recruitment and data collection.

OTHER COMMENTS

Please clarify how each research question is linked to the objective/s.

'Before and after study design' is not a study design. There are pre-and post-studies within the context of an observational or randomised controlled trial. Please clarify/revise.

Page 7, lines 39-42: As for health professionals, please briefly clarify what these surveys are before describing them in the data collection section.

Page 8, lines 5-7: This section is about data collection but it seems to be post-training only. Please clarify.

Page 8: 14-15: A trajectory analysis is appropriate only if there are at least three data points. If one cannot guarantee data for all three time points it might be better to rephrase this sentence and suggest alternative ways to visualise the data.

Page 8, Lines 21- 24 & 26-37: This information should be included in some other sections because they do not pertain to parental self-efficacy and sleep diaries (presumably, the outcome measures are obtained from sleep diaries in which case could you please add this information?). For example, lines 21-24 is about maternal and infant characteristics and lines 30-37 is about data analysis.

In some places the participant is a family and in some places the caregiver and in some places parents or caregivers. Please clarify throughout the paper. Also, please specify if it is multiple or single caregiver for a child.

Page 8: It is given that caregivers in the intervention group will be invited to a semi structured interview. Does this mean that there is a comparative group? Please clarify.

Please describe how health professionals were identified and recruited based on working with families experiencing the effects of living in deprivation and specify the definition of deprivation in this context.

Page 8, lines 16-17: Sleep diary should be a recording of daily activities. Therefore, please specify what data will be collected in these diaries and at what time points. Also, if it is daily data collection then please add how these data will be processed and analysed.

Please add a separate section on data analysis that includes both quantitative and qualitative analysis plan.

VERSION 1 - AUTHOR RESPONSE

Response to reviewer's comments

**Reviewer 1:**	
The study demonstrates a well-researched background and benefits from an experienced research team. I believe the qualitative methodology is adequately described.<br>Sample size calculation is well described. The estimated sample size ranges from 120-200 participants, with the final number depending on healthcare professional recruitment and response rates. While 180 approaches the upper limit, you should anticipate achieving approximately 160 participants, which remains statistically adequate. There are some points with regard to the quantitative part that requires more details.	Thank you for your comments.
Could you clarify the distribution plan for the baby sleep resources? Will these materials be provided to all participating families, both before and after the intervention period.	Thank you we have added "*We will ask health professionals to include caregivers who have not previously seen the resources, so as not to contaminate the pre-training data collection, and we will allow time for a short gap before introducing the resources. Where pre-training caregivers have completed both surveys, health professionals will be able to use the new resources with them if they wish to do so."*
The quantitative data collection methodology requires further detail, particularly regarding the timing of measurements at 3-4 weeks	We have added the following to the section headed Parental self-efficacy measures and sleep diaries:

and 8-10 weeks postpartum. Please specify:<br>- Whether responses should reflect the day immediately before the survey or participants should provide an average from the previous week etc.	"We will also collect data on: maternal age, parity, smoking/vaping during pregnancy, NICU admission, baby's sex, birthweight, partner support, partner smoking status, usual infant sleep location/position and sleep location/position last night (reflecting the night immediately before the survey)"<br>We have also included a copy of the survey as supplementary file A.
How should instances of non-supine or hazardous sleeping positions be recorded? Should the data reflect an average over the week prior to the survey? Will the two outcomes—non-supine sleeping and hazardous sleeping—be documented separately or combined into a single measure?	We have included a copy of the survey as a supplementary file, which shows that instances of non-supine or hazardous sleep situations will be recorded in either "usual" sleep or "last night", across two time points, closer to the intervention and a few weeks later. We have added: "Parental surveys will be downloaded and anonymised and analysed to investigate differences in infant care practices relating to the intervention between pre and post training. We will investigate whether hazardous co-sleeping or non-supine sleeping as a usual practice or "last night" changes between the two groups. These distinct measures will be documented separately. We will also compare parental self-efficacy between the two groups. As these surveys will be completed by caregivers of different babies, we will look closely at any differences in the characteristics of these individuals. Any characteristics that significantly differ between the groups will be adjusted for in the analysis. "
What methods will be implemented to ensure baseline characteristics are comparable between intervention and control groups so that any observed differences in outcomes can be attributed to the intervention.	We have included:<br>"We will encourage health professionals to use the new resources primarily with caregivers who are eligible for extra support, *with the same eligibility as before, though they will be different caregivers due to the baby's age requirement."*
How will the missing /unanswered/survey non-responses be handled. Has the non-response/lost to follow up been considered in the sample size?	Thanks we have added:<br>"We expect minimal loss to follow up due to the voucher incentive for the second survey and will over recruit slightly on the first survey (up to 95) to minimise this risk. Due to the long recruitment period we will have time to monitor follow up rates and adjust accordingly, if required." And "Survey responses to our main outcome data will be mandatory within the survey software."

There is no specific detail provided on how data will be recorded. Will the data/some of the data initially be documented in case notes and later transferred to an electronic system? What is the established procedure for ensuring data anonymization, as well as maintaining its quality and integrity throughout the process?	We have clarified this: "Surveys will be completed online (using JISC online survey platform), but can also be completed over the phone with a member of the research team" and "All data will be held on University of Bristol servers with access only available to the research team, and analysed using Stata statistical software.[36]<br>And "Parental surveys will be downloaded and anonymised"
**Reviewer 2:**	
The evaluation of an intervention for infant safe sleep is important. The proposed study by Pease et al involves intervention both at the level of healthcare professionals and parents/caregivers. The intervention includes web-based resources for health professionals and families to support infant safe sleep – The Sleep Planner, Safer sleep milestones card for babies on neonatal units and videos for caregivers and training video for healthcare professionals. However, the protocol description would benefit from some clarifications and additions described below.	Thank you for these comments.
It is appropriate that the sample size for the qualitative aspect of the study is based on pragmatic and feasible reasons. However, unless this is an exploratory/pilot study it is not appropriate to not calculate sample size for the quantitative aspect of the study. The sample size may not be adequate for the self-efficacy measure. Since the study is in-progress, could you please report the power calculation for comparison of responses before and after the intervention, and proportional differences in outcome measures between 4 and 8 weeks and also for the subgroup analyses and for comparison between sites?	Thank you, we have added: "Using 90% power and 5% significance, proportional differences between 13% and 24% will be detected (depending on the prevalence of the variable) using 90 respondents before and 90 respondents after the study. A much larger definitive study is planned but these findings will at least give us reasonable confidence intervals of the differences to help generate a future sample size calculation."
Please add details about how health care professionals' data from NoMAD questionnaires will be analysed. Besides this, please add information about how the analysis will account for within-site clustering effect. That is, please specify what method will be used to account for within-site clustering when reporting proportional differences in outcome measures between 4 and 8 weeks and for the subgroup analysis by risk score categories.	Thank you we have clarified this: "These questionnaires be analysed to look for differences between roles, regions, and changes across each timepoint. Petal charts will allow us to visualise these findings."

The pre-intervention activities seem to be unclear. Page 7- lines 16-17: Please clarify what pre-training data collection includes and please clarify when the training will take place with respect to recruitment.	We have expanded the pre-training data collection section to make this clearer, and added more detail to the intervention activities section: "When each *health professional has sent us details for at least 5 caregivers, or where we have reached the target of 90 caregivers completing the first survey*. " and "*The training will be completed online, on a dedicated password protected webpage, with full access to the online training video, the Baby Sleep Planner, animations, downloadable copies of the safer sleep milestones cards, and downloadable flyers with QR codes for the Baby Sleep Planner and animations. We will also post hard copies of these flyers to health professionals individually upon completion of the training. Health professionals will receive a certificate of completion for the training for their portfolio. " and "We will ask health professionals to invite caregivers who have not previously seen the resources, so as not to contaminate the pre-training data collection, and we will allow time for a short gap before introducing the resources, to reduce this risk. Where pre-training caregivers have completed both surveys, health professionals will be able to use the new resources with them if they wish to do so."*
Please clarify why data will be collected at two time-points for caregivers. What is expected to change during this period?	We have added:" *Two time-points will provide evidence of any sustained effects from the intervention, and covers the period of greatest risk for SIDS, according to the most recent data.*"
Please clarify what will be done with the data from caregivers collected during the pre-training period. Presumably, these are not the same caregivers after the training. Therefore, it would be important to compare the characteristics of these and if they differed that could account for the pre-post difference in intervention on the outcome measure instead of the intervention itself.	I hope we have answered this above, as this was also raised by reviewer 1.
The study is in-progress. Therefore, please describe the progress made other than recruitment. For example, it seems that the NoMAD questionnaire should have been completed by those recruited so far. Please provide details of recruitment and data collection.	We have updated the setting and context section to report details of sites recruited so far. We will report details of our recruitment and data collection in our paper reporting the findings, as these are not yet available, we have updated the recruitment end date to reflect this.

Please clarify how each research question is linked to the objective/s.	We feel that the following section explaining the development of our initial program theory clearly sets out how the objectives are designed to meet the aims within each research question. We have added further clarification in this section: "By using both qualitative and quantitative methods we hope to be able to generate data for these configurations, which relate specifically to our research questions."
'Before and after study design' is not a study design. There are pre-and post-studies within the context of an observational or randomised controlled trial. Please clarify/revise.	We have updated this in the Design section.
Page 7, lines 39-42: As for health professionals, please briefly clarify what these surveys are before describing them in the data collection section.	We have added in this detail: "about completing two surveys about their baby's sleep."
Page 8, lines 5-7: This section is about data collection but it seems to be post-training only. Please clarify.	We have added which data are collected pre or post, or both to each title to make this clearer. The only pre-training data collected is for parental self-efficacy and sleep diaries.
Page 8: 14-15: A trajectory analysis is appropriate only if there are at least three data points. If one cannot guarantee data for all three time points it might be better to rephrase this sentence and suggest alternative ways to visualise the data.	Thank you we have clarified this: "These questionnaires be analysed to look for differences between roles, and changes across each timepoint. Petal charts will allow us to visualise these findings."
Page 8, Lines 21- 24 & 26-37: This information should be included in some other sections because they do not pertain to parental self-efficacy and sleep diaries (presumably, the outcome measures are obtained from sleep diaries in which case could you please add this information?). For example, lines 21-24 is about maternal and infant characteristics and lines 30-37 is about data analysis.	We have re-named this section to "Parental Surveys" as this then includes maternal characteristics. We have moved the section on data analysis to the appropriate section, thank you.
In some places the participant is a family and in some places the caregiver and in some places parents or caregivers. Please clarify throughout the paper. Also, please specify if it is multiple or single caregiver for a child.	We have changed "families" to "caregivers" where relevant. We have added "One parent or caregiver per child can be included." To the section on participants.
Page 8: It is given that caregivers in the intervention group will be invited to a semi structured interview. Does this mean that there is a comparative group? Please clarify.	We are only interviewing caregivers who have had exposure to the intervention, as the focus of these interviews is a realist exploration of the context, use, and impact of the resources.

Please describe how health professionals were identified and recruited based on working with families experiencing the effects of living in deprivation and specify the definition of deprivation in this context.	We have clarified this: "Sites were recruited based on recent data showing regions with higher than average unexplained infant death rates.[1]" and "We will approach teams of health visitors, neonatal nurses, and specialist nurses (family nurse partnerships) working in regions with higher than average rates of unexplained infant mortality and invite them to take part in our evaluation."
Page 8, lines 16-17: Sleep diary should be a recording of daily activities. Therefore, please specify what data will be collected in these diaries and at what time points. Also, if it is daily data collection then please add how these data will be processed and analysed.	We have renamed these "sleep surveys" as it is more accurate. We have included a copy of the full survey as a supplementary file, which shows the sleep related questions.
Please add a separate section on data analysis that includes both quantitative and qualitative analysis plan.	We have expanded the section on "integration of data sources and analysis" to include this information, as it was also requested by reviewer 1, so is detailed above.
